# Addressing health disparities in hispanic communities through an innovative team-based medical spanish program at the medical school level – a single-institution study

**DOI:** 10.1186/s12909-022-03151-x

**Published:** 2022-02-14

**Authors:** Michael Oliver, Taylor Fernberg, Paul Lyons, Sambandam Elango, Gordon J. Green, Zohray M. Talib

**Affiliations:** California University of Science and Medicine, Colton, CA USA

**Keywords:** Medical Spanish, Medical Spanish program, Hispanic health, Disparities, Spanish OSCE, Spanish interpreters, Team-based learning, Language, Language evaluation

## Abstract

**Background:**

There are insufficient Spanish-speaking physicians to effectively serve a large and rapidly growing Spanish-speaking patient population.

**Methods:**

A team-based hybrid medical Spanish program was designed and implemented at a single medical school in Southern California. This pilot program consisted of a weekly in-person portion where students reviewed Spanish vocabulary and grammar and practiced clinical encounters in teams through active role play. Students supplemented in-class learning with online coursework. Program success was measured through physician-evaluated clinical encounters with Spanish-speaking standardized patients, a 100-question multiple-choice exam, and pre- and post-program surveys.

**Results:**

97% of students in the program (*n* = 32) received a passing grade at program completion. Student surveys demonstrated enthusiasm and engagement in weekly sessions (95% overall attendance, 97% reported feeling either excited or ready to learn prior to class). In a post-program survey, 100% of students felt better suited and increased desire to treat Hispanic patients. Additionally, all students indicated an interest in the continued use of Spanish in both their schooling and future practice. In a follow-up survey after three months of clinical experience in their 3^rd^ year of medical school, 100% of students reported that medical Spanish is "very beneficial" in patient care and that students with medical Spanish proficiency have advantages over non-speaking students when it comes to patient care opportunities. 100% felt that time spent learning medical Spanish during pre-clinical years was time well spent and that the medical Spanish program enhanced their care of Spanish-speaking students.

**Conclusions:**

The results of the pilot program show a significant increase in the ability of students to engage in clinical interaction in Spanish. The results of our study demonstrate a significant increase in the knowledge, clinical skills, and self-reported confidence of students to treat Hispanic patients. Furthermore, all students not only felt better equipped and more confident to treat Hispanic patients, but they also had an increased desire to do so moving forward in their careers. We conclude that an effective medical Spanish program can be executed simultaneously with a pre-clinical medical school curriculum.

**Supplementary Information:**

The online version contains supplementary material available at 10.1186/s12909-022-03151-x.

## Background

The Hispanic population continues to grow at a rapid rate throughout the United States [1]. This growth has not been paralleled by physicians trained to treat this large and mostly Spanish-speaking patient population [2, 3]. Language facilitates the connection between patients and physicians, and effective communication is critical to building trust and putting the patient at ease. The breakdown of communication that occurs between English-only physicians and Spanish-only patients leads to adverse outcomes for those patients [4–6]. Technology and medical interpreter services have attempted to bridge these disparities but continue to provide suboptimal care [7–9]. In addition to proven clinical care deficiencies encountered through a need for interpretation, these services create an added burden on the health system through increased patient encounter time [10–12].

In this period where there has been a spotlight placed on racial inequities, it is crucial that the medical field take the lead with tangible changes to patient care for minority communities. To critically address the inequitable care we currently provide for Hispanic patients, we must create programs that effectively train physicians who will work in communities with these growing patient populations. This will not only enhance medical care but will also create better community leaders.

With increasing demands on medical education curricula, advocating for additional time to learn Spanish is challenging. Our innovative program was designed with feasibility and sustainability in mind. The aim of our study is to assess the effectiveness of our team-based hybrid approach at increasing language proficiency and desire to treat Spanish-speaking patient populations.

In discussing this paper, we acknowledge the use of the terms “Hispanic” and “Spanish-speaker” throughout this paper. Not all Spanish-speakers may identify as Hispanic, and conversely, not all self-identified Hispanics speak Spanish. Both terms have been used throughout the paper, with “Hispanic” referring to persons of Latin-American descent, regardless of primary language, and “Spanish-speakers” referring to persons whose primary language is Spanish.

## Methods

### Program design

Our primary objective of the program was to enable clerkship students to communicate effectively in Spanish with their Spanish-speaking patients. Our secondary objective was to increase students’ desire to treat this population as future physicians.

The program took place on the campus of California University of Science and Medicine (CUSM) from May of 2019 (the end of the MD students’ 1^st^ year) through January of 2020, with a total of 7 months of in-class instruction.

An announcement was made to the entire MD class of 2022 regarding the structure and goals of the program. All but three of the students (who were already fluent in Spanish) were eligible for participation in the program. Students were told that completion of the program would not appear on their transcript, with no penalty for dropping or failing the program. However, successful completion would be mentioned in their Dean’s letter.

The program was designed as a team-based hybrid model, whereby in-class instruction was supplemented by an online course [13]. The online course, Canopy Medical Spanish, consisted of 22 lessons revolving around basic Spanish grammar. These were to be completed alongside the in-class material. Each week, students attended a 1-hour in-class team-based learning session. They were expected to complete an assigned online lesson in Canopy prior to attending the session. Canopy was used as a means for giving students instruction in Spanish grammar, while the in-class portions focused mostly on learning and practicing words and phrases related to the medical interview. Students were also provided with a copy of a textbook as an additional resource [14]. For in-class sessions, students were separated into teams of four, based on pre-program Spanish fluency and gender. Students were also deliberately placed in teams different from their learning communities in our MD program. Each team of four was led by a Spanish-speaking tutor. The tutors were comprised of Spanish-speaking students, staff, and standardized patients at CUSM. Spanish proficiency of tutors was self-reported as fluent. To assure equitable delivery of content from tutors to groups, weekly meetings were held with tutors. These meetings included a discussion of the delivery of the previous week’s content, the strengths and weaknesses of each group, and which content would be delivered in the following class.

Following the teaching of basic patient interview content and skills (Introductions/greetings, opening and closing the patient interview), the bulk of the course was taught through a systems-based approach (e.g., MSK, GI, Cardiovascular, Endocrine, etc.). The layout for the medical aspects of the program was unique in that it ran parallel to the medical school curriculum so that as they were learning medical material in English, they would learn that same material in Spanish. The teaching guidelines for their end-of-program objective structured clinical examination (OSCE) largely followed that of the clinical skills curriculum at CUSM with modifications made to address the core aspect of the program – to train students to communicate effectively with Spanish-speaking patients.

Each weekly class consisted of three aspects: grammar, vocabulary, and a patient interview. Grammar activities and worksheets were created by program leadership and completed in-class with Spanish tutors present to answer questions and assess for understanding. These activities combined topics covered in the Canopy online course with the system that would be covered in the in-class session, allowing for a more cohesive experience for students between the online and in-class portions. Weekly vocabulary lessons were delivered through team-based interactive competitive activities. Students practiced their patient interview skills via weekly role plays with other students and Spanish-speaking standardized patients. Cases for the role plays were associated with the medical content (systems-based) being learned at the time and were created by students and tutors. A Spanish-fluent facilitator was present during each weekly session to ensure timely, organized, and equitable delivery of the lesson. In addition to their weekly session, each student was provided an opportunity to complete multiple mid-program mock patient encounters with standardized patients to track their progress within the program and receive feedback.

### Analysis of program

Our evaluation approach was based on the three levels of assessment from the Kirkpatrick Model: student experience, student knowledge, and student behavior [15]. These were assessed through three surveys, three assessments in an online course, a multiple-choice exam, and an OSCE.

Students took a survey at the start and end of the program to gauge their overall experience with the program. A third survey was administered to the students during their third year to determine the degree to which they use their newly acquired Spanish in their daily patient encounters. Surveys were created using SurveyMonkey. A link to the surveys was emailed to each student to be completed at their convenience. Students were told that the survey would be anonymous and not connected to identifying student information. Surveys are listed in their entirety in Additional files 1.

As described in methods, students completed lessons from Canopy Medical Spanish alongside the in-class materials. As part of the online course, they were required to pass a level 1 and a level 2 exam. The final ten lessons and level 3 exam are to be completed during their clinical rotations.

The multiple-choice exam consisted of 100 questions written by our program leaders based on topics covered in class and Canopy (Table 1). The exam was administered through Examsoft.

**Table 1:** Topics covered on the multiple-choice end of program exam, by percentage

Topic	% of test
Vocabulary	40%
Regular Present Tense	10%
Imperative Tense	10%
Irregular Present Tense	5%
Progressive Tense	5%
Perfect Tense	5%
Preterit Tense	3%
Imperfect Tense	3%
Preterit vs. Imperfect	3%
Ser vs. Estar	2%
Por vs. Para	2%
Near Future	2%
Direct and Indirect Objects	2%
Articles	2%
Adjectives	2%
Possessive Pronouns	2%
Tener que	2%

OSCEs consisted of two 12-minute patient encounters, each with a native Spanish-speaking standardized patient and performed entirely in Spanish. In one encounter, students obtained a focused history and performed a physical exam. In the other, students obtained a full history. After each encounter, students recounted in English all the information they gathered and provided a differential diagnosis. Spanish-speaking physicians graded students based on their general language skills, medical Spanish, and the overall patient interaction. In addition to an overall percentage score, evaluators rated students on their ability to interact with a Spanish-speaking patient on a scale from 0-9 (see Additional file 2 for full rubric and scale description).

## Results

Out of 61 eligible students from the CUSM 2022 class, 39 expressed interest and attended the first class in May of 2019, and of these, 32 completed the program. For those completing the program, 97% received a passing grade. Attendance was 95% overall across all students and class sessions. Statistical analysis of the results was performed using IBM SPSS Statistics, version 26 (IBM, Armonk, New York).

### Student experience

When asked how they felt at the beginning of class each week, 47% indicated excitement, and 50% indicated a readiness to learn. With regards to the program content, 72% of students indicated in-class sessions were the most beneficial aspect of program, 16% of students answered “Canopy”, and 12% “Other.” Additionally, all 32 students agreed they would have benefited from additional classroom time with a teacher.

The students’ expectations of the program were also assessed. When asked to what degree their Spanish-speaking improved compared to their expectations at the start of the program, all students felt they had improved at least as much as they had expected, with 75% of students expressing they had improved more than they expected.

### Student knowledge

The multiple-choice exam was graded based on the raw percentage of questions that students answered correctly. Scores ranged from 67%-100%, with a mean score of 90.8% (8.9) and a median of 94.0%.

OSCE evaluations consisted of both a raw score and an evaluator-rated ability of the student to interact with the Spanish-speaking patient. All but one student passed (>70%) the OSCE, with a mean of 83% (7.7). In terms of the ability to interact with a Spanish-speaking patient, the mean rating for 32 students was 5.2 (1.3) on a scale from 0-9.

**Fig. 1:**
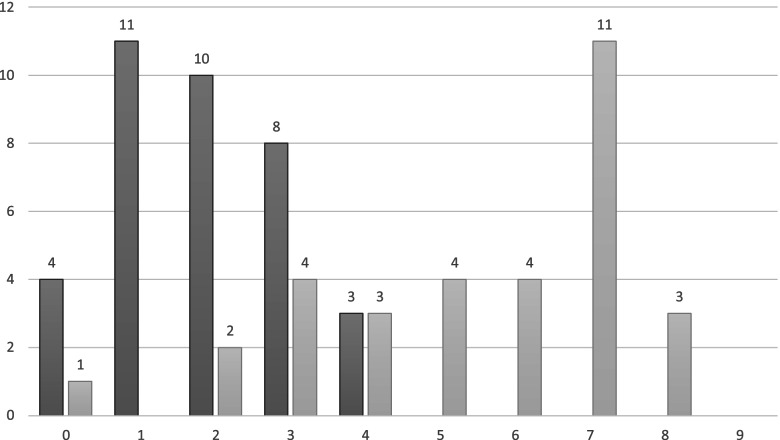
From 0-9, a self-rated ability of students to interact with a Spanish-speaking patient. Dark bars represent student ratings from a survey administered prior to the start of the program, while light bars represent those from a survey administered after the program

With regards to self-rated ability to interact with a Spanish-speaking patient, there were 36 respondents for the beginning-of-program survey, with a mean self-rated ability of 1.9 (1.2). 32 students completed the end of program survey, reporting a mean self-rated ability of 5.4 (2.1) (see Table 2 for detailed description and results). A comparison between pre- and post-program self-ratings can be found in Fig. 1.

**Table 2:** Pre/post-program surveys: Self-rated ability of students to interact with a patient

Rate your ability to interact with a patient:	Pre-program	Post-program
0 - I don't speak any Spanish	4	1
1 – I am limited to greetings and goodbyes	11	0
2 – I can understand very common medical terminology but wouldn't feel comfortable responding or initiating conversation in Spanish.	10	2
3 – With difficulty, I can speak to patients about very common topics and common anatomy.	8	4
4 – With relative ease, I can speak to patients about very common topics and common anatomy.	3	3
5 – With difficulty, I can speak to patients about more intricate medical and nonmedical terminology.	0	4
6 – With relative ease, I can speak to patients about more intricate medical and nonmedical terminology.	0	4
7 – With very limited help or while making clinically insignificant mistakes, I can conduct an entire patient interaction (history & physical).	0	11
8 – I can conduct an entire patient interaction without the aid of a translator.	0	3
9 – I consider myself a fluent Spanish-speaker.	0	0

### Student behavior

100% of students in the post-program survey indicated an increased desire to treat Hispanic patients in addition to feeling better suited to do so. 100% also indicated an interest in the continued use of Spanish in both their schooling and future practice.

When asked, “How likely are you to use your Spanish abilities during your clinical rotations?” the mean value for 32 respondents was 3.5 (0.7) on a scale from 0-4. When asked to identify the degree to which they hope to use Spanish in their future career, 100% indicated some degree of planned Spanish use, with 94% indicating they hope to use Spanish without requiring translation services.

There were 26 respondents to the third-year survey, a response rate of approximately 84%. The complete results of the survey can be found in Table 3. 100% of students felt that this medical Spanish program has enhanced their care of Spanish-speaking patients, making them feel more confident speaking to Spanish-speaking patients. All students indicated that when confronted with a Spanish-speaking patient, they would at least sometimes attempt to speak Spanish, with a majority (58%) indicating that they "always" attempt to speak Spanish.

**Table 3:** Third-year clinical survey results

		#	Percentage
Based on your experience after 3+ months in the clinical setting, how beneficial is medical Spanish in patient care?	Very beneficial	26	100%
	Somewhat beneficial	0	0%
	Not beneficial	0	0%
In retrospect, do you believe spending time learning medical Spanish during the pre-clinical years is time well spent?	Yes	26	100%
	No	0	0%
Do you believe students with medical Spanish proficiency have advantages over non-speaking students regarding patient care opportunities?	Yes	26	100%
	No	0	0%
How would you compare overall patient care provided by a proficient medical Spanish provider vs. telephone interpreter services?	Both are equally as good	0	0%
	Both provide equal quality; telephone services are not as efficient	8	31%
	Proficient medical Spanish provider provides higher quality of care	18	69%
How frequently do you encounter Spanish-speaking patients?	Every day	15	58%
	3-4 times per week	9	35%
	1-2 times per week	1	4%
	1-2 times per month	1	4%
	Never	0	0%
When faced with a Spanish-speaking patient, how often do you attempt to speak Spanish?	Always	15	58%
	Most of the time	6	23%
	Sometimes	5	19%
	Rarely	0	0%
	Never	0	0%
Compared to prior to the medical Spanish course, do you feel more confident speaking to Spanish-speaking patients?	Yes	26	100%
	No	0	0%
Do you think the medical Spanish course has enhanced your care of Spanish-speaking patients?	Yes	26	100%
	No	0	0%

All 26 respondents indicated that medical Spanish is "very beneficial" in patient care, agreeing further that students with medical Spanish proficiency have advantages over non-speaking students when it comes to patient care opportunities. Additionally, all 26 students believed the time spent learning medical Spanish during pre-clinical years was time well spent.

When compared to telephone interpreter services, 69% of students felt that a proficient medical Spanish provider provides a higher quality of care, and 31% of students indicated that although they feel telephone services provide equal quality, the telephone services are not as efficient. Through serving a large Spanish-speaking population at their home teaching hospital, they have had ample exposure to telephone interpreter services, and their resultant interpretation of those experiences forms the basis for the claims made above about the quality of those services.

## Discussion

Based on student and evaluator ratings, students in the pilot program showed a significant increase in their ability to engage in clinical interaction with Spanish-speaking patients in a controlled setting. The results of our study indicate an increase in the knowledge, clinical skills, and self-reported confidence of students to treat Hispanic patients. Furthermore, all students not only felt better equipped and more confident to treat Hispanic patients, but they also had an increased desire to do so – both during rotations and moving forward in their careers. Of note, this paper reflects preliminary findings as the 3^rd^ survey was conducted only three months into their clinical curriculum; long-term follow-up studies are to come.

It is clear from our study that medical students can make significant strides toward the enhancement of their language and communication skills by engaging in a language program in their pre-clinical years. Furthermore, we were able to provide the framework for a program that motivated student attendance and engagement in an optional program. We conclude that an effective medical Spanish program can be executed simultaneously with a pre-clinical medical school curriculum.

Seven students dropped from the program due to time constraints. However, the majority of students in our program were able to perform well academically and unanimously expressed high value in dedicating time towards pre-clerkship medical Spanish (Table 2). Recent news that Step 1 of the United States Medical Licensing Exam will be moving to a pure pass/fail grading system is likely to result in increased motivation of students to work on other aspects of professional development, including but not limited to the acquisition of language in the pursuit of expanding clinical reach and decreasing health inequities [16].

The success of the pilot program was recognized by faculty and students and has prompted a second cohort of the program. The 2^nd^ iteration started in July 2020 with an enrollment of 140 students and includes lengthening the program, increased weekly focus on conversational Spanish with a native speaker, and addition of biweekly meetings with a clinical upper-class mentor to discuss cases and practice Spanish use in the clinical setting.

One limitation to our study is a lack of externally validated tools for measuring Spanish proficiency. Although exams from the online course Canopy Medical Spanish were used, the rest of the exams, including the OSCE, were created internally. The OSCE rubric was taken from an existing, validated rubric at CUSM and then modified to address the goals of the program; notably the ability to complete clinical encounters with Spanish-speaking patients. However, following the modifications, the rubric did not undergo any additional external validation for evaluating proficiency prior to being implemented. In future studies, all evaluation tools will go through the assessment and evaluation committee prior to implementation. Furthermore, we would like to acknowledge that all OSCE exams, regardless of language, have limitations in the sense that they are staged encounters where standardized patients have pre-planned responses and students are aware of the grading rubric. In this sense, students may be able to prepare for such an encounter in a way that they would not be able to for a real patient encounter. With regard to Spanish, the OSCE may not be able to simulate the speed, volume, intonation, accent, or variation of the Spanish-language that participants might encounter in clinical practice.

A significant challenge faced in the development of the program was creating a cohesive experience for the students between the online and in-class portions. We wanted the in-class sessions to not only teach new content related to the medical interview but also to reinforce the concepts learned from the online course. This was accomplished by tailoring each week’s session to include systems-related grammar activities, which coincided with the systems being covered for the medical interview. Creating these activities was one of the most difficult aspects of the program creation.

Another difficulty we faced was ensuring that the delivery of content from tutors was standardized and equitable between groups. This was particularly difficult due to the lack of Spanish-speaking faculty and students. Given that the fluency of the tutors was only determined through self-report, the standardization of teaching became especially important for maintaining intervention fidelity. It was for this reason that we held weekly meetings with tutors as described in our methods section.

## Conclusions

The successful implementation of a medical Spanish program during pre-clinical years of medical school curricula should be replicated, especially in schools located in densely populated Spanish-speaking communities like ours. Widespread development of programs such as the one described here can change the landscape of medicine through the quality of care we provide for Hispanic communities and the perception of the medical field within Hispanic communities. It should be noted that this program and other programs alike are meant to add value to pre-existing resources (translator services) rather than detracting from or replacing them. Furthermore, we believe physician language competency will be beneficial not only in clinical settings but also in non-clinical settings within the community, where physicians will not have access to translator services. Future research should examine the efficacy, educational cost, and healthcare benefit of scaling a Medical Spanish program to include a greater number of participants. Long-term studies should be conducted to discern the impact of Spanish-language acquisition on patient care and how this affects pre-existing translator services. Furthermore, future studies should explore the integration of Medical Spanish as a core component of medical school curricula at institutions serving Spanish-speaking populations.

## Supplementary Information

**Additional file 1.** Student surveys (pre-, post-, and third-year)

**Additional file 2.** Medical Spanish OSCE Rubric

## Data Availability

All data generated or analyzed during this study are included in this published article [and its supplementary information files]

## Abbreviations

CUSM: California University of Science and Medicine
GI: Gastrointestinal
MSK: Musculoskeletal
OSCE: Objective Structured Clinical Examination

## References

[1] Noe-Bustamante LLM, Krogstad JM. Hispanic population surpassed 60 million in 2019, but growth has slowed. Pew Research Center. 2020; [Available from: https://www.pewresearch.org/fact-tank/2019/07/08/u-s-hispanic-population-reached-new-high-in-2018-but-growth-has-slowed.

[2] Language Spoken at Home [Table]. United States Census Bureau; 2019 [updated 12/20/2020. Available from: https://data.census.gov/cedsci/table?q=Language%20Spoken%20at%20Home&tid=ACSST1Y2019.S1601.

[3] Sanchez G, Nevarez T, Schink W, Hayes-Bautista DE (2015). Latino Physicians in the United States, 1980-2010: A Thirty-Year Overview From the Censuses. Acad Med.

[4] Divi C, Koss RG, Schmaltz SP, Loeb JM (2007). Language proficiency and adverse events in US hospitals: a pilot study. Int J Qual Health Care.

[5] Sadanand A, Ryan MH, Cohen S, Ryan MS (2018). Development of a Medical Spanish Curriculum for Fourth-Year Medical Students. PRiMER.

[6] Pippins JR, Alegria M, Haas JS (2007). Association between language proficiency and the quality of primary care among a national sample of insured Latinos. Med Care.

[7] Flores G, Abreu M, Olivar MA, Kastner B (1998). Access barriers to health care for Latino children. Arch Pediatr Adolesc Med.

[8] Manson A (1988). Language concordance as a determinant of patient compliance and emergency room use in patients with asthma. Med Care.

[9] Rivadeneyra R, Elderkin-Thompson V, Silver RC, Waitzkin H (2000). Patient centeredness in medical encounters requiring an interpreter. Am J Med.

[10] Baker DW, Hayes R, Fortier JP (1998). Interpreter use and satisfaction with interpersonal aspects of care for Spanish-speaking patients. Med Care.

[11] Fagan MJ, Diaz JA, Reinert SE, Sciamanna CN, Fagan DM (2003). Impact of interpretation method on clinic visit length. J Gen Intern Med.

[12] Ngo-Metzger Q, Sorkin DH, Phillips RS, Greenfield S, Massagli MP, Clarridge B (2007). Providing high-quality care for limited English proficient patients: the importance of language concordance and interpreter use. J Gen Intern Med.

[13] The NIH-Awarded Medical Spanish Syllabus - CanopyLearn: Canopy Innovations; [Available from: https://withcanopy.com/medical-spanish-syllabus.

[14] Pilar Ortega M. Spanish and the Medical Interview A Textbook for Clinically Relevant Medical Spanish. 2 ed: Elsevier; 2015 07/06/2015. 512 p.

[15] Kirkpatrick JD (2016). Kirkpatrick's Four Levels of Training Evaluation.

[16] White CB, Fantone JC (2010). Pass-fail grading: laying the foundation for self-regulated learning. Adv Health Sci Educ Theory Pract.

