# Correction to: Addressing health disparities in Hispanic communities through an innovative teambased medical spanish program at the medical school level – a single-institution study

**DOI:** 10.1186/s12909-022-03195-z

**Published:** 2022-04-01

**Authors:** Michael Oliver, Taylor Fernberg, Paul Lyons, Sambandam Elango, Gordon J. Green, Zohray M. Talib

**Affiliations:** California University of Science and Medicine, Colton, CA USA

**Correction to: BMC Med Educ 22, 98 (2022)**

**https://doi.org/10.1186/s12909-022-03151-x**

Following publication of the original article [1], we have been informed that the Supplementary information was incorrectly uploaded.

The original article has been corrected.
