# Supplementary material for: Addressing health disparities in hispanic communities through an innovative team-based medical spanish program at the medical school level – a single-institution study
Source: BMC Med Educ. 2022 Feb 14;22:98. doi: 10.1186/s12909-022-03151-x (PMC8845388; doi:10.1186/s12909-022-03151-x)
Supplement: Supplementary file 1 — Additional file 1. Student surveys (pre-, post-, and third-year) [file 12909_2022_3151_MOESM1_ESM.pdf]

Student surveys (pre-, post-, and third-year)

Pre-course survey

1.) Please indicate the class you are in:
    a. Beginner
    b. Intermediate
2.) Indicate how you feel (heading into Medical Spanish class each week?) when you are preparing for Medical Spanish to start:
    a. Excited
    b. Ready to learn
    c. Indifferent
    d. Stressed
    e. I regret signing up
3.) On a scale from 1 to 5, with 1 being extremely disappointed and 5 being extremely happy, how happy are you with:
    a. In-class sessions to date
    b. Your team
4.) Based on my involvement in the Medical Spanish course so far, by the end of this course, I am confident that my Spanish will improve:
    a. Substantially more than expected
    b. More than expected
    c. About as much as expected
    d. Less than expected
    e. Substantially less than expected
5.) How would you rate your knowledge, understanding, and ability to interact with Hispanic culture, on a scale from "Lacking" (1) to "Fully enriched" (5)?
6.) What are the strengths of this course?
7.) What would you change about this course?
8.) What would make tutors more helpful?
9.) Prior to beginning this course, rate your ability to interact with a patient:
    a. 0 – I don't speak any Spanish
    b. 1 – I am limited to greetings and good-byes.
    c. 2 – I can understand very common medical terminology but wouldn't feel comfortable responding or initiating conversation in Spanish.
    d. 3 – With difficulty, I can speak to patients about very common topics and common anatomy.
    e. 4 – With relative ease, I can speak to patients about very common topics and common anatomy.
    f. 5 – With difficulty, I can speak to patients about more intricate medical and nonmedical terminology.

g. 6 – With relative ease, I can speak to patients about more intricate medical and nonmedical terminology.
h. 7 – With very limited help or while making clinically insignificant mistakes, I can conduct an entire patient interaction (history & physical).
i. 8 – I can conduct an entire patient interaction without the aid of a translator.
j. 9 – I consider myself a fluent Spanish-speaker.

10.) Using the same scale, please indicate where you expect to be after taking this course.

11.) Using the same scale, please indicate where you would like your ability level to be.

Post-course survey

1.) Please indicate the class you are in:
    a. Beginner
    b. Intermediate
2.) Rate your ability to interact with a patient:
    a. 0 – I don't speak any Spanish
    b. 1 – I am limited to greetings and good-byes.
    c. 2 – I can understand very common medical terminology but wouldn't feel comfortable responding or initiating conversation in Spanish.
    d. 3 – With difficulty, I can speak to patients about very common topics and common anatomy.
    e. 4 – With relative ease, I can speak to patients about very common topics and common anatomy.
    f. 5 – With difficulty, I can speak to patients about more intricate medical and nonmedical terminology.
    g. 6 – With relative ease, I can speak to patients about more intricate medical and nonmedical terminology.
    h. 7 – With very limited help or while making clinically insignificant mistakes, I can conduct an entire patient interaction (history & physical).
    i. 8 – I can conduct an entire patient interaction without the aid of a translator.
    j. 9 – I consider myself a fluent Spanish-speaker.
3.) To what degree did your Spanish-speaking ability improve relative to your expectations at the start of the course?
    a. Substantially less than expected
    b. Less than expected
    c. About as much as expected
    d. More than expected
    e. Substantially more than expected
4.) Which portion of the curriculum was most beneficial to you:
    a. In-class sessions
    b. Canopy
    c. Other
5.) Do you think you would have benefited from additional classroom time with a teacher focusing on language/grammar principles and general conversational Spanish (not specific to medicine)?
    a. Yes
    b. No
6.) Has this course encouraged you to continue to develop your Spanish-speaking abilities moving forward in your medical career?
    a. Yes

    b. No

7.) How likely are you to use your Spanish abilities during your clinical rotations?
    a. 0 – I don't plan to use my Spanish during rotations
    b. 1
    c. 2
    d. 3
    e. 4 – I plan to speak with all patients with a Spanish-speaking preference

8.) After this course, how do you hope to use Spanish in your future practice as a physician? (long-term aspirations)
    a. I don't foresee myself using Spanish in patient encounters.
    b. I hope to use Spanish in a limited capacity with patients while requiring translation services to complete interactions.
    c. I hope to have a strong enough skill set to treat Spanish-speaking patients without compromising medical care.
    d. I hope to become certified to treat Spanish-speaking patients

9.) Following the course, do you feel better suited to treat Hispanic patients?
    a. Yes
    b. No

10.) Do you feel that this course has increased your desire to treat Hispanic patients?
    a. Yes
    b. No

Third-year survey

1. Based on your experience after 3+ months in the clinical setting, how beneficial is medical Spanish in patient care?
    a. Very beneficial
    b. Somewhat beneficial
    c. Not beneficial
2. In retrospect, do you believe spending time learning medical Spanish during the pre-clinical years is time well spent?
    a. Yes
    b. No
3. Do you believe students with medical Spanish proficiency have advantages over non-speaking students regarding patient care opportunities?
    a. Yes
    b. No
4. How would you compare overall patient care provided by a proficient medical Spanish provider vs. telephone interpreter services?
    a. Both are equally as good
    b. Both provide equal quality, telephone services are not as efficient
    c. Proficient medical Spanish provider provides higher quality of care
5. How frequently do you encounter Spanish-speaking patients?
    a. Every day
    b. 3-4 times per week
    c. 1-2 times per week
    d. 1-2 times per month
    e. Never
6. When faced with a Spanish-speaking patient, how often do you attempt to speak Spanish?
    a. Always
    b. Most of the time
    c. Sometimes
    d. Rarely
    e. Never
7. Compared to prior to the medical Spanish course, do you feel more confident speaking to Spanish-speaking patients?
    a. Yes
    b. No
8. Do you think the medical Spanish course has enhanced your care of Spanish-speaking patients?
    a. Yes
    b. No
