# Supplementary material for: Addressing health disparities in hispanic communities through an innovative team-based medical spanish program at the medical school level – a single-institution study
Source: BMC Med Educ. 2022 Feb 14;22:98. doi: 10.1186/s12909-022-03151-x (PMC8845388; doi:10.1186/s12909-022-03151-x)
Supplement: Supplementary file 2 — Additional file 2. Medical Spanish OSCE Rubric [file 12909_2022_3151_MOESM2_ESM.pdf]

Medical Spanish OSCE Rubric

Name of Student ________________________________ Date Completed:

Setting the stage of the encounter	NOT Performed	Incompletely Performed	Performed
1. Greetings and introduction (introduces self with the name and as a medical student)	❑	❑	❑
2. Disinfects hands prior to shaking hands with patient	❑	❑	❑
3. Verifies identifying data & Determines how the patient wishes to be addressed	❑	❑	❑
4. Ensures the patients' privacy and comfort	❑	❑	❑
5. Sets the agenda (explains what will happen during the encounter)	❑	❑	❑
6. Asks permission to proceed	❑	❑	❑

Patient history	Did not perform	Incompletely Performed	Performed Moderately Well	Performed Exceptionally
1. Elicits **chief complaint** (uses open ended question such as "What brings you in"	❑	❑	❑	❑
2. Gives the patient the opportunity to relay the issues in his or her own words (e.g. asks "What more can you tell me about that?"	❑	❑	❑	❑
**3. History of presenting illness (HPI).** ❑ Site ❑Onset ❑ Character ❑Radiates ❑ Aggravating ❑ Alleviating ❑ Severity	❑	❑	❑	❑
4. HPI : Elicited **case specific questions** ❑ ❑ ❑ ❑ ❑	❑	❑	❑	❑
5. Inquires about **past medical history** ❑ Previous Illness ❑ Surgery ❑ Hospitalization ❑ Trauma / Accidents	❑	❑	❑	❑
6. Inquires about ❑ medications ❑ drug allergies ❑ food allergies ❑ environmental allergies ❑ immunizations	❑	❑	❑	❑
7. Elicits **habits** ❑ alcohol ❑ tobacco ❑ drugs	❑	❑	❑	❑
8. Elicits other aspects of **the social history** ❑ marital status ❑sexual history ❑ diet ❑ exercise ❑ employment ❑ hobbies ❑travel ❑pets ❑sick contacts	❑	❑	❑	❑
9. Elicits **family history** ❑ parents ❑ siblings / children	❑	❑	❑	❑
**10. Review of Systems** ❑ Fever / Weight changes ❑ Neuro ❑ Cardio ❑ Respiratory ❑ GI ❑ Renal ❑ MSK	❑	❑	❑	❑
**11. Summarizes and checks for accuracy of information provided**	❑	❑	❑	❑

CLOSING THE ENCOUNTER AND PROFESSIONALISM	Did not perform	Incompletely Performed	Performed
1. Informs about what happens next	❑	❑	❑
2. Asked the patient "Does he/she has any questions? Concerns?"	❑	❑	❑
3. Demonstrated attentiveness via non-verbal body language	❑	❑	❑
4. Used appropriate language and avoided medical jargon	❑	❑	❑
5. Demonstrated professional behavior	❑	❑	❑
6. Makes a logical differential diagnosis	❑	❑	❑

Medical Spanish OSCE Rubric

Name of Student ___________________________________ Date Completed:

Setting the stage of the encounter	NOT Performed	Incompletely Performed	Performed
1. Greetings and introduction (introduces self with the name and as a medical student)	❑	❑	❑
2. Disinfects hands prior to shaking hands with patient	❑	❑	❑
3. Verifies identifying data & Determines how the patient wishes to be addressed	❑	❑	❑
4. Ensures the patients' privacy and comfort	❑	❑	❑
5. Sets the agenda (explains what will happen during the encounter	❑	❑	❑
6. Asks permission to proceed	❑	❑	❑

Patient history	Did not perform	Incompletely Performed	Performed Moderately Well	Performed Exceptionally
1. Elicits **chief complaint** (uses open ended question such as "What brings you in"	❑	❑	❑	❑
2. Gives the patient the opportunity to relay the issues in his or her own words (e.g. asks "What more can you tell me about that?"	❑	❑	❑	❑
**3. History of presenting illness (HPI).** ❑ Site ❑Onset ❑ Character ❑Radiates ❑ Aggravating ❑ Alleviating ❑ Severity	❑	❑	❑	❑
4. HPI : Elicited **case specific questions** ❑ ❑ ❑ ❑ ❑	❑	❑	❑	❑
**5. Review of Systems** ❑ Fever/weight changes ❑ Neuro ❑ Cardio ❑ Respiratory ❑ GI ❑ Renal ❑ MSK	❑	❑	❑	❑
**6. Summarizes and checks for accuracy of information provided**	❑	❑	❑	❑
7. Asks patient to lie down ❑	❑	❑	❑	❑
8. Asks patient to expose stomach ❑	❑	❑	❑	❑
9. Inspection: Names at least 3 things they are looking for: ❑ ❑ ❑	❑	❑	❑	❑
10. Tells patient they will listen for bowel sounds ❑	❑	❑	❑	❑
11. Tells patient that they will tap on their stomach ❑	❑	❑	❑	❑
12. Tells patient they will perform light palpation ❑ Asks patient if they feel pain ❑	❑	❑	❑	❑
13. Tells patient they will perform deep palpation ❑ Asks patient if they feel pain ❑	❑	❑	❑	❑
14. Tells patient they will press down (on McBurney's point) ❑	❑	❑	❑	❑
15. Asks patient to lift leg against their hand (Psoas sign) ❑	❑	❑	❑	❑
16. Tells patient that they will press down ❑ Asks the patient to breathe deeply ❑	❑	❑	❑	❑

Medical Spanish OSCE Rubric

EVALUATOR AND PATIENT ASSESSMENT OF SPANISH ABILITY	Language is a barrier to encounter	Significant effect on encounter	Moderate effect on encounter	Minor effect on encounter	No negative effect on encounter
1. Rate the student's speaking ability (rate, fluidity, pronunciation)	❑	❑	❑	❑	❑
2. Rate the student's use of medical vocabulary (quantity of words and use of follow-up questions/comments)	❑	❑	❑	❑	❑
3. Rate the student's ability to use grammar correctly	❑	❑	❑	❑	❑
**4. Rate the student's ability to understand the patient**	❑	❑	❑	❑	❑
5. (Patient interpretation) Did the patient understand the student?	❑	❑	❑	❑	❑
6. (Patient interpretation) Did the student demonstrate attentiveness and respect through verbal and non-verbal communication?	❑	❑	❑	❑	❑

	Check only one box below
Using the scale below, rate the student's ability to interact with a patient:	
0 – Doesn't speak any Spanish	❑
1 – Is limited to greetings and goodbyes.	❑
2 – Can understand very common medical terminology but wouldn't feel comfortable responding or initiating conversation in Spanish.	❑
3 – With difficulty, can speak to patients about very common topics and common anatomy.	❑
4 – With relative ease, can speak to patients about very common topics and common anatomy.	❑
5 – With difficulty, can speak to patients about more intricate medical and nonmedical terminology.	❑
6 – With relative ease, can speak to patients about more intricate medical and nonmedical terminology.	❑
7 – With very limited help or while making clinically insignificant mistakes, can conduct an entire patient interaction (history & physical).	❑
8 – Can conduct an entire patient interaction without the aid of a translator.	❑
9 – I consider this student a fluent Spanish-speaker.	❑

***Narrative Feedback** (Comment on the overall performance – communication skills, examination skills, technique)*

Medical Spanish OSCE Rubric
